# Appearance traits in fish farming: progress from classical genetics to genomics, providing insight into current and potential genetic improvement

**DOI:** 10.3389/fgene.2014.00251

**Published:** 2014-08-04

**Authors:** Nelson Colihueque, Cristian Araneda

**Affiliations:** ^1^Laboratorio de Biología Molecular y Citogenética, Departamento de Ciencias Biológicas y Biodiversidad, Universidad de Los Lagos, OsornoChile; ^2^Laboratorio de Biotecnología y Genética Aplicada a la Acuicultura, Departamento de Producción Animal, Facultad de Ciencias Agronómicas, Universidad de Chile, SantiagoChile

**Keywords:** skin pigmentation, body shape, appearance traits, fish farming, quantitative trait loci

## Abstract

Appearance traits in fish, those external body characteristics that influence consumer acceptance at point of sale, have come to the forefront of commercial fish farming, as culture profitability is closely linked to management of these traits. Appearance traits comprise mainly body shape and skin pigmentation. Analysis of the genetic basis of these traits in different fish reveals significant genetic variation within populations, indicating potential for their genetic improvement. Work into ascertaining the minor or major genes underlying appearance traits for commercial fish is emerging, with substantial progress in model fish in terms of identifying genes that control body shape and skin colors. In this review, we describe research progress to date, especially with regard to commercial fish, and discuss genomic findings in model fish in order to better address the genetic basis of the traits. Given that appearance traits are important in commercial fish, the genomic information related to this issue promises to accelerate the selection process in coming years.

## INTRODUCTION

Over the past few decades, body shape and skin pigmentation have become valuable appearance traits in commercial fish (**Table 1**). Due to increasing market sophistication, fish size, meat quality, and other traditional traits are not the only attributes that influence consumer choice at point of sale, especially when fish are sold whole.

**Table 1:** Examples of appearance traits for body shape and skin pigmentation used in fish farming.

Category of trait	Species	Strains	Trait characteristic	Practical application	Reference
Body shape	Common carp (*Cyprinus carpio*)	Aischgrund and Galician	High-backed	To increase esthetics of body shape	Ankorion et al. (1992)
		var. *wuyuanensis* (also called Purse red carp)	Broadly elliptical body (red skin); standard length to body height ratio: 2.3	To increase esthetics, creating a desirable “wallet” shape and all-red body color	Zhang et al. (2013)
		var. *haematopterus* (also called Amur wild carp)	Spindle-shaped body (steel-gray skin); standard length to body height ratio: 3.5	To increase desirability for sport fishing (its body is elongated, making it an excellent sport fish)	Zhang et al. (2013)
	Rainbow trout (*Oncorhynchus mykiss*)	Finnish national breeding program	Slender body (i.e., low body height to length ratio; silvery skin with fewer spots)	To produce fish more visually appealing desirable for the whole carcass market	Kause et al. (2003)

Skin color	Tilapia (*Oreochromis niloticus*)	Red strains (red Stirling, red Yumbo, etc)	Red body color without signs of normal black pigmentation of wild-type fish	To add value to the final product	McAndrew et al. (1988), Moreira et al. (2005)
	Rainbow trout (*Oncorhynchus mykiss*)	Blue Back	Intense bluish back; whitish belly; reduced number of dark spots, both on the back and below the lateral line	To satisfy market demands	Colihueque et al. (2011)

On the subject of skin pigmentation, previous work conducted in other livestock species has demonstrated that proper handling of pigmentation traits allows for response to consumer demands for various food products, such as skin color in pigs and egg shell, yolk, and skin color in chickens (see review by Hudon, 1994). This topic is relevant for producers because the color of a food product is a quality attribute for the consumer. For example, consumers perceive redder salmon filets as being fresher, better-tasting, and higher quality as compared with paler salmon, and, therefore, they are willing to pay significantly more for the product (Anderson, 2000; Alfnes et al., 2006). Therefore, to satisfy modern market demands and increase profitability, producers are forced to manage external traits more intensively on an industrial scale, in particular body shape and skin color.

However, this is not an easy task, because body shape and skin color in fish are complex traits, involving numerous genetic and environmental factors. Thus, progress in this field will depend in part on dissecting the underlying genetics of these traits for future implementation of modern selection strategies, such as marker-assisted selection based on molecular data.

**FIGURE 1:**
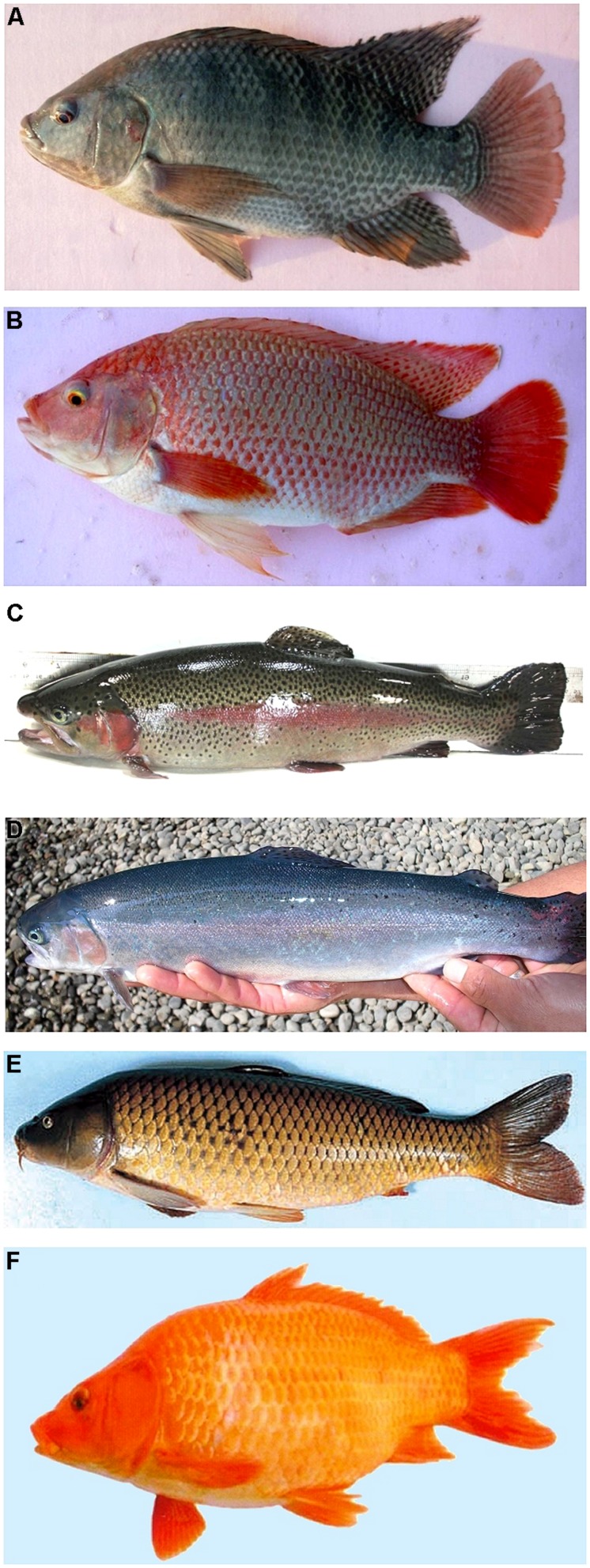
**Examples of commercial fish strains with improved skin pigmentation and body shape. (A)** A wild-type tilapia (*Oreochromis niloticus*) with normal black pigmentation; **(B)** a red strain tilapia (red Yumbo) with improved red skin pigmentation; **(C)** a wild-type rainbow trout (*Oncorhynchus mykiss*) with normal pigmentation and **(D)** a Blue Back rainbow trout with improved intense bluish back, whitish belly, and a reduced number of dark spots; **(E)** a common carp (*Cyprinus carpio*) of var. *haematopterus* (or Amur wild carp) with a spindle-shaped body and steel-gray skin color; and **(F)** a common carp of var. *wuyuanensis* with improved broadly elliptical body (red skin color).

In commercial fish, such as the common carp, tilapia, sea bream, and salmonids (Pillay and Kutty, 2005), this strategy will complement progress made to date based solely on breeding values estimated with phenotypic and genealogical information or classical genetics which, for example, has enabled the development of new strains (**Figure 1**).

Further understanding of this issue may be gained from progress achieved in model and ornamental fish, where characterization of the inheritance mode of mutation, genes, and quantitative trait loci (QTL) for external traits is more advanced.

In this review, we describe efforts made to improve the external traits in commercial fish based on classical genetic approaches, as well as recent progress in genomics, the latter initially aimed at identifying the specific region that harbors genes controlling quantitative traits. This information, together with data available on this issue in model fish, will enhance progress in this field, an objective of tremendous importance for producers who need to increase the competitiveness of their cultures by managing external characteristics that give added value to cultured fish.

## FISH BODY SHAPE

In common carp, one of the first domesticated fish in the world (Balon, 2004), the long process of domestication has produced a domesticated phenotype very different from the wild-type phenotype (Ankorion et al., 1992; Zhang et al., 2013). Many of these changes have arisen due to intentional selection of traits, but it is equally true that many traits are the result of an unintentional selection process. This phenomenon emerged as a result of the adaptation of fish to captive conditions, quite different than the natural environment inhabited by wild-type fish.

The domesticated phenotype phenomenon arises because the body shape of an organism results from the integration of morphological, behavioral, and physiological traits (Reid and Peichel, 2010), where different genetic and environmental pressures can lead to functional trade-offs (Reznick and Ghalambor, 2001; Ghalambor et al., 2003; Walker, 2010). This creates functional constraints, where those changes with the greatest positive and fewest negative effects on fitness will be selected (Reid and Peichel, 2010). For example, in natural populations, there is a relationship between body shape and swimming performance, but body shape is also influenced by foraging behavior, the risk of predation, and stream velocity (Webb, 1984; Walker, 1997). The trade-off for body shape also operates in captive populations. For instance, cultured populations of rainbow trout selected for rapid growth result in more rotund fish, given the existence of a positive genetic correlation of body mass with body shape and condition factor (Gjerde and Schaeffer, 1989; Kause et al., 2003); that is, mass gain in fish achieved by increasing body width and height rather than by increasing body length.

It has been shown that other factors, such as water velocity (Pakkasmaa and Piironen, 2001), rearing environment (Swain et al., 1991), fish density, and diet (Higgs et al., 1992; Einen et al., 1998; Jenkins et al., 1999) may also modify body shape in fish. This phenomenon occurs given that many morphological growth-related traits show phenotypic and genetic correlations in fish (Kause et al., 2003; Martyniuk et al., 2003), whose origins are related to the genetic architecture of traits such as covariation in QTL location or conservation of chromosomal regions homologous across species (Reid et al., 2005).

In common carp, this process has produced various phenotypes of commercial value that are currently used in fish farming, such as the high-backed and elliptical body shape morphs, typical of the Galician and Wuyuanensis strains, respectively (Ankorion et al., 1992; Zhang et al., 2013). Even this process of domestication can reach a high level of body shape modification, such as it has been observed in the ornamental goldfish (*Carassius auratus*), where various morphological traits have been modified (e.g., body shape, fins, and eyes). Several of these modified traits can be found in the same individual, giving rise to popular strains called “monstrosities” (Balon, 1990).

Alterations of morphology characteristics, mainly body length and fins (Haffter et al., 1996) can also be seen in mutants of zebrafish (*Danio rerio*). In the guppy (*Poecilia reticulate*), variation in male body shape occurs in association with mating success (Tripathi et al., 2009). Therefore, available evidence in fish indicates that these organisms are highly amenable to morphological modification, already widely explored in ornamental as well as in model fish.

The underlying genetics of phenotypic variation is beginning to be understood in various commercial fish (Massault et al., 2009; Loukovitis et al., 2013; Zhang et al., 2013). These studies are focusing on finding significant QTL for morphometric traits based mostly on geometric morphometry; in these analyses, different types of molecular markers have been used. These investigations are contributing to our understanding of the genetic architecture of divergence in body shape, by means determining the number of genes or QTLs that contribute to a particular trait, or the number of traits that a particular gene or QTL affects, i.e., the pleiotropic effect, and the location of genes or QTLs within the genome that affect body shape, along with their interaction.

Progress in model fish should be mentioned here, particularly in zebrafish (Haffter et al., 1996) where a set of dominant Mendelian loci affecting body shape and fins in induced mutants have been identified. For example, loci that affect body shape may cause a reduction of overall body length in the adult fish, due to a reduction either in the length of vertebrae (*stöpsel* mutant) or number of vertebrae (*däumling* mutant). Interestingly, mechanisms of body shape variation involving axial length modification also occur naturally across several fish species (Ward and Mehta, 2010), which indicates that this mechanism has been of evolutionary significance for body form differentiation in fish.

Recent work on QTL searching in commercial fish clearly supports the existence of major genes underlying the quantitative genetic variation of morphological and body shape-related traits (**Table 2**). In Gilthead seabream (*Sparus aurata*), Loukovitis et al. (2013), using half-sib regression analysis, found significant morphology QTLs, e.g., distances from pectoral fin to dorsal fin or from pectoral fin to anal fin (see **Table 2**), in three linkage groups (9, 21, and 25) identified at genome-wide level that explain 18.5 to 27.1% of trait variation. This result suggests the existence of one locus in each linkage group affecting several traits in this fish. Moreover, given that QTLs affecting body weight were located at the same positions for the linkage groups 9 and 21 (Loukovitis et al., 2011), the authors conclude that there might be only one pleiotropic QTL in each LG affecting overall body size. This is in accordance with the high genetic correlations (r_G_ > 90%) observed between all traits analyzed (see **Table 2**), including body weight. These results, combined with those obtained from previous studies (Boulton et al., 2011), underline highly significant loci affecting overall morphology in *S. aurata*.

**Table 2:** Examples of QTLs for body shape-related traits mapped on different commercial fish genomes.

Species	Trait	Linkage group (LG) where QTLs were detected. Name of QTL (bold), linked markers, and respective phenotypic variation noted in parentheses	Reference
Gilthead seabream (*Sparus aurata*)	Standard length, body length, pectoral dorsal 1, pectoral dorsal 2, body length 2, pectoral anal 2, pectoral anal 1, dorsal fin length, belly length, body depth 2, body depth 3, head length	LG-9 (**SL**,** BL**,** PecDor1**, **Pecdors2**,** BL2**,** PectAnal1**,** PecAnal2**, Bd20, 18.5–23%)LG-21 (**DFL**, **BellyL**, **BD2**, **BD3**, ELD36 and SAGT1, 18.6–27.1%)LG-25 (**HL**, 22.6%)	Loukovitis et al. (2013)

Sea bass (*Dicentrarchus labrax*)	Combination of morphometric traits (standard length, head length, body length, pre-anal length, abdominal length, post-anal length, head depth, body depth)	LG-1B (**MORPH**, 14%)LG-4 (**MORPH**, 13%)LG-6 (**MORPH**, 9.4%)LG-7 (**MORPH**, 16%)LG-15 (**MORPH**, 12%)LG-24 (**MORPH**, 13%)	Massault et al. (2009)

Common carp (*Cyprinus carpio* var. *haematopterus*)	Body height Body width Standard length	LG-1 (**qbh1**, snp0163, 20.4%; **qbw1**, snp0163, 20.7%) LG-12 (**qsl12**, snp0315, 21.1%; **qbh12**, snp1133, 18.9%) LG-20 (**qbh20**, hlj1717, 19.5%)	Zhang et al. (2013)


*QTLs were detected at genome-wide level using permutation tests at a significance threshold value of *P* < 0.05 for S. *aurata* and D. *labrax*, and of *P* < 0.01 for C. *carpio*.*

On the other hand, using half-sib regression analysis and variance component analysis at the genome-wide level in sea bass (*Dicentrarchus labrax*), six significant QTLs for a combination of morphometric traits (standard length, head length, body length, pre-anal length, abdominal length, post-anal length, head depth, body depth; see **Table 2**) on linkage groups 1B, 4, 6, 7, 15, and 24 were reported by Massault et al. (2009). These QTLs explain between 9.4 and 16% of phenotypic variance. In this study, a body weight QTL was discovered at the same linkage groups (linkage groups 4 and 6) and at similar positions as morphology QTLs, which might explain the high correlation observed between body weight and all morphometric traits studied in this fish.

Moreover, in common carp (*Cyprinus carpio*), in a primary genome-wide scan using single nucleotide polymorphisms (SNPs) and microsatellite markers, Zhang et al. (2013) found five significant QTLs for body-shape related traits (body height, body width and standard length) located at linkage groups 1, 12, and 20, which explain 20.4 to 20.7%, 18.9 to 21.1%, and 19.5% of phenotypic variance, respectively. Given that QTLs of linkage group 1 were located in the same interval, it was concluded that only one QTL produced pleiotropic effects on these traits, which was not the case for QTLs found in linkage group 12, indicating that different factors control the traits. Importantly, this study provides strong evidence that the marked body shape differences of *Cyprinus carpio* populations, in particular between *Cyprinus carpio* var. *wuyuanensis* and *Cyprinus carpio* var. *haematopterus*, depend on quantitative genetic variations that control different body shape-related traits that may have originated through the process of selective breeding that has occurred for decades in this species.

In salmonids, another important group of commercial fish (Pillay and Kutty, 2005), progress has been made in this field through QTLs searching, mainly for growth-related traits, including fork length, body weight, and Fulton’s condition factor, and also for meristic traits (for review see Araneda et al., 2008). For example, in Atlantic salmon (*Salmo salar*), four QTLs for condition factor and two for body weight were detected in comparative studies with rainbow trout (*Oncorhynchus mykiss*) and Arctic charr (*Salvelinus alpinus*). One strong QTL explaining 20.1% of variation in body weight was found on linkage group AS-8, while another QTL with a strong effect on condition factor accounting for 24.9% of trait variation was found on linkage group AS-14. This result suggests that a significant portion of quantitative variation in body weight and condition factor in this species is under the control of a few QTLs with relatively large effects.

However, to date no study has been specifically undertaken to search QTLs in salmonids based on the geometric morphology method. It is noteworthy that in some species of this group, such as rainbow trout, a marked intra- and inter-population differentiation in body shape has been observed (Kause et al., 2003; Hecht et al., 2012; Pulcini et al., 2013). For example, Pulcini et al. (2013), in a common-garden experiment, found a marked morphological variation in body shape traits such as body profile, head length, dorsal and anal fin length, and caudal peduncle size, using geometric morphometry among wild, semi-wild and domestic lines of this species. Domestic lines have a deeper body profile, with longer dorsal and anal fins and shorter and deeper caudal peduncles than wild lines. This differentiation, attributed to exposure of domestic lines to captive conditions, suggests that the variations may result from fixed genetic differences among lines due to the existence of QTL. Therefore, further QTL analysis in rainbow trout would be useful in clarifying the underlying genetics of this striking differentiation in body shape. To achieve this goal the use of SNPs it is possible given that these markers are considered to be the most desirable molecular markers for developing high-density genome scan to discover and locate target genes underlying the quantitative traits (Wang et al., 1998). This approach has been demonstrated to be efficient to discover several QTLs in guppy that control the complex patterns of skin pigmentation of males (Tripathi et al., 2009). However, it needs to use next-generation sequencing analysis to discover thousands of such SNPs (Miller et al., 2007; Davey et al., 2011) and in several cases developing SNP chips to perform genome-wide scans.

## SKIN PIGMENTATION

The cellular basis of skin pigmentation in fish is well known. Skin color depends on five types of pigment cells (or chromatophores) known as melanophores, xanthophores, erythrophores, iridophores, and leucophores, each producing a different color (black or brown, yellow or orange, red, iridescent, blue, silver or gold, and white, respectively; Fujii, 1969, 1993). The underlying genetics of skin pigmentation phenotype, however, has been explored mostly for qualitative traits by means of large-scale analyses of natural or induced color mutants, mainly in model fishes (see review by Colihueque, 2010). Evidence from studies in these and in other fish species indicates that the inheritance mode of qualitative traits for skin pigmentation has a simple genetic basis (Tave, 1986), i.e., a monogenetic control, which may be recessive, completely/incompletely dominant, and co-dominant or sex-linked. Moreover, these studies indicate that several genes participate in producing a specific skin color or color pattern that may be involved in chromatophore development, pigment synthesis, and pigment expression. For example, about 90 and 40 genes of this type have been identified in zebrafish and medaka (*Oryzias latipes*), respectively, that control specification, proliferation, survival, differentiation, and distribution of chromatophores, among other processes.

However, recent studies emphasize that skin pigmentation in fish can also possess a more complex genetic architecture, characterized by specific genome regions that harbor genes controlling quantitative traits (**Table 3**). For example, in the threespine sticklebacks (*Gasterosteus aculeatus*), two significant QTLs on linkage groups 1 and 6 that control the degree of barring and explain 26.6% of the variance of the trait were found (Greenwood et al., 2011). Given that these QTLs were associated with spatial variation in melanophore number (linkage group 6) and degree of melanization of melanophores (linkage group 1), this finding reveals the existence of different loci underlying variation in pigment patterns of this fish, which shows striking diversity of among freshwater (barred) and marine (unbarred) populations. Moreover, the number of dorsal and ventral melanophores is also controlled by different loci in this fish, since they were mapped to linkage group 7 and linkage group 1, respectively.

**Table 3:** Examples of QTLs for skin pigmentation traits mapped on different fish model genomes.

Species	Trait	Linkage group (LG) where QTLs were detected. Name of QTL (bold), linked markers, and respective phenotypic variation noted in parentheses	Reference
Threespine sticklebacks (*Gasterosteus aculeatus*)	Degree of barring Degree of melanization Number of dorsal melanophores Number of ventral melanophores	LG-1 (**barring**, chrI:3310077, 6.6%) LG-1 (**melanization**, chrI:4816374, 11.7%) LG-6 (**barring**, chrVI:15780594, 20%) LG-1 (**ventral melanophores**, chrI:21909727, 8.9%) LG-7 (**dorsal melanophores**, chrVII:1728753, 11.6%)	Greenwood et al. (2011)

Guppy (*Poecilia reticulate*)	Dorsal fin black area Dorsal fin orange area Central blue white spot Anterior main black stripe Anterior orange spot Black spot by gonopodium Central orange spot Posterior main black stripe Posterior ventral black stripe Posterior orange spot Hind fin lower orange area	LG-1, LG-2, LG-4, LG-9, LG-12 (**Dorsal fin black area**, 22.9%) LG-1, LG-2, LG-4, LG-6, LG-7, LG-16 (**Dorsal fin orange area**, 26.8%) LG-2, LG-12, LG-17, LG-21, LG-23 (**Central blue white spot**, 26.5%) LG-8, LG-19 (**Anterior main black stripe**, 9.4%) LG-6, LG-7, LG-9, LG-12, LG-20 (**Anterior orange spot**, 23%) LG-01, LG-04, LG-07, LG-12, LG-16 (**Black spot by gonopodium**, 19.1%) LG-05, LG-08, LG-20, LG-22 (**Central orange spot**, 15.3%) LG-04, LG-10, LG-12, LG-13, LG-22, LG-23 (**Posterior main black stripe**, 21.6%) LG-01, LG-02, LG-13, LG-16, LG-18 (**Posterior ventral black stripe**, 17.7%) LG-08, LG-09, LG-15, LG-16, LG-18 (**Posterior orange spot**, 26.2%) LG-03, LG-04, LG-06, LG-12, LG-20 (**Hind fin lower orange area**, 23.3%)	Tripathi et al. (2009)

QTLs were detected at genome-wide level using permutation tests at a significance threshold value of *P* < 0.05 for G. *aculeatus* and P. *reticulate*.

Through synteny analysis, Greenwood et al. (2011) identified the *Gja5* gene contained in the barring QTL on linkage group 6, which encodes a gap junction protein whose mutation disrupts the normal pigmentation pattern in zebrafish, in which spots form in place of the typical horizontal stripes, caused by an altered melanophore distribution (Watanabe et al., 2006). Moreover, the region on linkage group 1 that mapped QTLs associated with both barring and degree of melanization contains the *tyrosinase* gene, which encodes a key enzyme in melanin synthesis, whose mutation eliminates all pigmentation in zebrafish and medaka (Koga et al., 1995; Iida et al., 2004). Therefore, these results suggest that a few genes with large effects underlie the pigmentation pattern variation in the threespine sticklebacks.

In guppy males, a more complex control of pigmentation pattern has been observed (Tripathi et al., 2009), including a phenotype characterized by multi-colored areas with an ornamental function involved in female choice and in male mating success, and therefore, important for male fitness. In the genome of this fish, using interval mapping and the multiple-QTL model, 49 QTLs for 11 areas of pigmentation traits were found (see **Table 3**), which explain 9.4 to 26.8% of phenotypic variation in these traits. In addition, these QTLs were mapped in 19 out of 24 linkage groups of this species, although mainly on linkage group 12 and 4. QTLs located on linkage group 12, which corresponds to the sex chromosome of the guppy, indicate that loci responsible for polymorphisms in guppy color patterns are clustered on this chromosome. This result coincides with previous knowledge regarding physical linkage of major color pattern loci to sex chromosomes in this species (Winge and Ditlevsen, 1947; Khoo et al., 1999). In summary, the results obtained in this fish strongly suggest that multiple QTLs with minor effects contribute to each color trait in guppy males.

In commercial fish, most color phenotypes of commercial value are qualitative traits known to be under Mendelian control, such as the Red Stirling strain of tilapia (*Oreochromis niloticus*; dominant inheritance, McAndrew et al., 1988), or the iridescent metallic blue variant of rainbow trout (recessive inheritance, Kincaid, 1975); therefore, given its simple inheritance mode, they could be more easily subjected to selective breeding for new stocks with particular colors.

However, there are some rainbow trout skin pigmentation phenotypes of commercial value, such as the Blue Back (Colihueque et al., 2011) and Finnish national breeding program (Kause et al., 2003) traits with complex pigmentation patterns comprising several attributes (skin color, number, size, and position of dark spots) that vary continuously at the intrapopulation level. A substantial quantitative genetic component for the different attributes that compose these traits has been reported (Kause et al., 2003; Díaz et al., 2011). As it has been seen in model fish, it is possible that these skin pigmentation traits may possess a complex genetic architecture, with the existence of a variable number of quantitative loci with a minor or major effect for the different trait attributes. Further analysis of these traits will clarify their particular genetic architecture.

## CONCLUDING REMARKS

In farmed fish, several traits are taken into account in order to obtain a quality fish harvest suitable for marketing. These traits include body shape and skin pigmentation, both of which affect consumer acceptance of marketed fish at the point of sale. A fish with an improved appearance has greater consumer acceptance and, therefore, has a higher sale value than a fish with a normal appearance.

There has been some progress in this area with commercial fish, including traditional and new cultures, mainly through selective breeding or classical genetic analysis. This selection strategy has resulted in new fish stocks whose market participation is constantly increasing, contributing to the improved profitability of fish cultures. This trend is expected to continue over the next few years due to the sophistication of the market in many areas of the world. Therefore, there is interest in fish selection to ensure specimens that are visually appealing, for example, tilapia, rainbow trout, common carp, gilthead sea bream, and sea bass.

However, to meet this challenge, fish farmers must adapt and align their selective breeding goals with market demands. One tool that may be explored to achieve this objective derives from the discovery of QTLs or genes that underlie body shape and skin pigmentation, in which continuous variation of the different attributes that compose these traits is usually observed. This information could be used to implement selective breeding based on molecular markers tightly linked to QTLs that control various appearance traits of commercial interest, that is, marker-assisted selection. This strategy may offer a more rapid response, yielding fish with a specific external appearance to satisfy market demands.

## Conflict of Interest Statement

The authors declare that the research was conducted in the absence of any commercial or financial relationships that could be construed as a potential conflict of interest.
